# The process of nurses’ confrontation with ethical conflicts in home care: a grounded theory study

**DOI:** 10.1186/s12912-025-04195-2

**Published:** 2025-12-22

**Authors:** Mostafa Gholami, Tahereh Najafi Ghezeljeh, Forough Rafii, Soodabeh Joolaee

**Affiliations:** 1 Student Research Committee, Nursing and Midwifery Faculty, Iran University of Medical Sciences, Tehran, Iran; 2 Department of Medical Surgical Nursing, School of Nursing and Midwifery, Iran University of Medical Science, Tehran, Iran; 3 School of Nursing and Midwifery, Iran University of Medical Sciences, Tehran, Iran; 4 Cardiovascular Nursing Research Center, Rajaie Cardiovascular Medical and Research Center, Iran University of Medical Sciences, Tehran, Iran; 5 Nursing and Midwifery Care Research Center, Health Management Research Institute, Iran University of Medical Sciences, Tehran, Iran; 6 Fraser Health Auhority, Vancouver, Canada

**Keywords:** Ethics, Conflict, Nursing, Grounded theory, Home-care services

## Abstract

**Background:**

Ethical conflicts (ECs) are an inseparable part of nursing care, particularly home care (HC). Nurses’ inability to effectively manage ECs may lead to occupational stress, job burnout, and low patient care quality. Despite various studies on ECs, there is limited research regarding the process of nurses’ confrontation with them in HC settings.

**Aim:**

This study aimed to explore the process of nurses’ confrontation with ECs in HC.

**Research Design:**

This qualitative study was conducted from February 2023 to August 2025 using Corbin and Strauss’s approach to grounded theory.

**Methods:**

Twenty-two unstructured and semi-structured interviews were held with sixteen nurses recruited through purposeful and theoretical sampling from HC centers in Tehran, Iran. Data were analyzed using Corbin and Strauss’s approach to grounded theory.

**Results:**

The main concern of participants was fear of harming patients, and their four strategies to manage it were expedient thinking, persuading, self-justifying, and disclosing. They used these strategies to balance the interests of all parties involved in the EC situation, including patients, families, colleagues, and HC center authorities. Therefore, the core category of the theory formulated in this study was “Balancing the interests”.

**Conclusion:**

Nurses’ confrontation with ECs in HC is a complex process influenced by a variety of contextual factors. Study findings can be used to develop purposeful educational and supportive programs for improving nurses’ ethical decision-making.

**Supplementary Information:**

The online version contains supplementary material available at 10.1186/s12912-025-04195-2.

## Introduction

Population changes, growing number of elderly people, increasing prevalence of chronic and debilitating diseases, and improvement of patients’ and families’ awareness of their rights have increased the use of home care (HC) services in healthcare systems [1, 2]. HC is a comprehensive and professional community-based care system, encompassing medical and caring services such as medication therapy, medical examinations, wound care, client education, complication management, physiotherapy, speech therapy, medication management, and client empowerment. It aims to support clients’ health and provide care in their living environment [3]. The prevalence of HC service use among elderly people is 10% in Sweden and 32% in China [4, 5]. Comparable rates have been reported in other countries, including 11.7% in Brazil [6] and 6.8% in Australia [7].

HC is suitable for clients who do not need hospital-based services but need strong support to have a safe life at home. This type of care cuts healthcare costs, reduces the workload of healthcare providers, and improves patients’ and their families’ mental and social outcomes and quality of life [8]. Meanwhile, HC presents nurses with complex responsibilities and various challenges. Examples include the inappropriateness of the home environment for care provision, problems with interdisciplinary collaboration, inadequate supervision of HC nurses’ practice, and communication-related issues [9, 10].

Ethical conflicts (ECs) are among the most significant challenges in HC for nurses [11]. ECs occur when nurses face two or more conflicting ethical principles or professional values, none of which is entirely justifiable. These conditions complicate decision-making and often lead to confusion, anxiety, or stress for nurses [12]. ECs usually arise around issues like privacy protection, confidentiality maintenance, patient autonomy, end-of-life decision-making, and conflicts between professional benevolence and clients’ preferences [10]. These conditions can cause nurses to experience problems such as ethical dilemmas, uncertainty, or ethical distress [12].

Various factors may contribute to ECs in HC settings. HC nurses spend considerable time with patients and their families and encounter diverse and complex sociocultural and familial contexts, which can be sources of ECs [9]. Moreover, family members’ serious engagement in care-related decision-making despite their low health literacy level, unclear professional boundaries, limited access to supportive and legal resources, and conflicts between nurses’ professional values and families’ cultural and religious expectations can contribute to ECs [13]. ECs that are not appropriately identified, analyzed, and managed can lead to negative consequences, including low job motivation, ethical distress, job burnout, low job satisfaction, low care quality, and endangered patient safety [12, 14].

The process of nurses’ confrontation with ECs is one of the determinants of the outcomes of ECs. A qualitative study on intensive care nurses showed that nurses used detachment and engagement as strategies to cope with ECs. The subthemes of these two main strategies were ignorance of ethical problems, seeking methods to express emotions, perspective-taking, and identification of positive assets [15]. Another study on community health nurses’ management of ECs also formulated the model of moral compassing, which states that nurses go through four phases to manage ECs. These phases were undergoing a visceral reaction, self-talk, seeking validation, and mobilizing support for action or inaction [16].

ECs are context-dependent [13, 17], and nurses’ confrontation with them is influenced by various factors like socioeconomic backgrounds, values, ethical beliefs, professional identity, religious-cultural competence, professional experience, organizational support, and sociocultural environment [18]. Although some previous studies explored nurses’ experiences of HC-related ECs [19, 20], they did not specifically address the context of these conflicts. Moreover, some of them focused on nurses’ confrontation with ECs in hospital settings [16]. Therefore, there are still knowledge gaps regarding nurses’ confrontation with HC-related ECs, particularly in societies with a religious context like Iran. In Iran, nurses are formally trained to make clinical and ethical decisions based on widely accepted principles, including autonomy, beneficence, non-maleficence, and justice. Nursing ethics courses at undergraduate and graduate levels, along with ethics-focused instruction during clinical practicums, provide the foundation for ethical competence. Ethical considerations are also reinforced in clinical settings through the Patient Rights Charter, supervisory reviews, ethics rounds, and continuing education programs. These educational and organizational frameworks shape how nurses recognize, interpret, and respond to ECs in practice. Thus, this study aimed to explore the process of nurses’ confrontation with ECs in HC.

## Methods

### Design

This qualitative study was reported in accordance with the Consolidated Criteria for Reporting Qualitative Research (COREQ) checklist to ensure comprehensive and transparent reporting(see Supplementary File) [21]. This qualitative study was conducted from February 2023 to August 2025 using Corbin and Strauss’s approach to grounded theory. ECs in HC are context-dependent phenomena influenced by social interactions among patients, families, and healthcare providers. Therefore, qualitative designs, particularly grounded theory, help explore their different aspects and provide a good understanding of nurses’ confrontation with them [18].

### Participants

Participants were sixteen nurses selected from HC centers in Tehran, Iran. Participants were eligible if they could communicate in Persian and were willing to provide informed consent. We attempted to select participants with maximum variation in terms of their age, gender, marital status, educational level, and HC experience to include a wide range of experiences in the study [19]. Access to potential participants was obtained through HC centers and their managers. After explaining the study objectives and presenting the official approval letter from the Vice-Chancellor for Research of Iran University of Medical Sciences, the supervisors were asked to introduce eligible nurses. The researcher then contacted these nurses by phone to provide further information and arrange interviews with those who agreed to participate. The interviews were conducted at the HC centers and at the nursing faculty, based on the participants’ preferences and mutual agreement. Three potential participants indicated that they did not have sufficient time to participate in the study and therefore declined. No participants withdrew after agreeing to take part. The first participant was an experienced and collaborative HC nurse selected through purposeful sampling [20]. Her characteristics helped us collect more in-depth data [22]. Other participants were selected through theoretical sampling, i.e., based on the results of simultaneous data collection and analysis. Theoretical sampling seeks to collect data based on the concepts and themes derived from the collected data [20].

### Data collection

The researcher (M. Gholami), who collected the data, was a PhD candidate in nursing at the time of the study. He had received formal training in qualitative research methods and interviewing techniques as part of his doctoral program. The first two interviews were unstructured to improve flexibility in data collection. These two interviews aimed at an in-depth exploration of participants’ experiences and identification of the important concepts related to EC situations. The questions of the semi-structured interviews were developed based on the existing literature, experts’ comments, and the results of unstructured interviews. An example of the interview questions was, “What did you do when you were uncertain about the ethical correctness of a care-related action or behavior?” Pointed questions were also used during the interviews based on participants’ responses to the main interview questions to collect more details about their experiences (Supplementary File 1). Six participants were interviewed twice to clarify unclear points from their first interviews. In total, 22 interviews were held with sixteen participants, and all of them were digitally recorded and immediately transcribed. The length of the interviews was 60–90 min, with a mean of 75 min. We terminated data collection after data saturation, i.e., when no new categories emerged from the interviews and no additional data were obtained to further develop the properties of the existing categories. The information obtained became repetitive and confirmed the previously developed concepts. The research team regularly discussed the ongoing analyses, and consensus was reached that further interviews would not contribute additional insights to the developing theory [19].

### Data analysis

Data analysis was conducted simultaneously with data collection based on the five-step approach recommended by Corbin and Strauss [22]. The five steps of this approach are open coding to identify concepts, developing concepts in terms of their properties and dimensions, analyzing data for context, bringing process into the analysis, and integrating categories.

#### Open coding to identify concepts

Interviews were audio-recorded by the researcher (M. Gholami) and transcribed immediately after completion. The transcripts were reviewed several times to obtain a general understanding of the underlying concepts. Then, meaning units were determined and coded, and the codes were grouped based on their similarities.

#### Developing concepts in terms of their properties and dimensions

In this step, constant comparison was performed to develop concepts in terms of their properties and dimensions. We compared the different parts of each interview with each other and also compared its codes with the codes of previous interviews, and grouped the codes based on their similarities. Then, categories were grouped into more abstract categories based on their properties and dimensions. This process was continued to fully develop all categories in terms of their properties and dimensions. All study authors participated in data analysis.

#### Analyzing data for context

In this step, which was taken concurrently with the previous step, we attempted to determine the structural and contextual factors that influenced nurses’ confrontation with ECs in HC. Accordingly, we used questions like “Where?”, “When”, and “Under what conditions” during data analysis to determine these factors in the data.

#### Bringing process into the analysis

In this step, we examined the constant patterns of actions, emotions, and interactions that participants used in their confrontation with ECs in HC. We frequently reviewed our memos, codes, and categories to determine the strategies and behaviors that they showed in response to their problems caused by contextual conditions. We developed a reflective coding matrix as well as questions like “What?”, “When?”, “Why?”, “How?”, and “With what consequences?” to ensure the accuracy of the generated categories, clearly describe them, and bring process into analysis [23].

#### Integrating categories

This step aimed to determine the core category and develop an integrated theoretical structure. We determined the core category, linked other categories to it, and revised the theoretical construct through carefully assessing the categories, writing storylines, reviewing the memos, and drawing diagrams.

We improved theoretical sensitivity through constant comparative analysis, memo writing, and reflective discussions within the research team. During coding and categorization, researchers continuously compared incidents within and across interviews to identify similarities, differences, and relationships between concepts. Analytic memos were written to capture emerging ideas and guide deeper conceptualization. The researchers’ professional backgrounds in nursing and familiarity with ethical issues in clinical practice further enhanced their theoretical sensitivity, enabling them to recognize subtle meanings and interpretations in participants’ experiences while avoiding premature closure or the imposition of preexisting theories. The research team also discussed the developing categories in several sessions to challenge interpretations and ensure that the analysis moved beyond mere description toward theoretical abstraction [22].

### Trustworthiness

Lincoln and Guba’s four criteria were used to ensure trustworthiness. These criteria are credibility, dependability, confirmability, and transferability [24]. Credibility was achieved through prolonged engagement with the data, member checking, peer checking, negative cases analysis, and triangulation. Based on the literature, member check can be conducted with some or all participants [25]. In our study, we performed member checking with four participants—who were key informants representing diverse experiences—were provided with a copy of the generated codes and were asked to comment on the congruence between their real experiences and the codes. In this process, parts of the polished analytical product, including the main findings, were returned to the participants to determine whether they accurately represented their experiences. Follow-up interviews were also conducted to ensure the accuracy of data analysis. Based on the participants’ feedback and suggestions for clarification, some codes were refined to enhance the accuracy and transparency of the findings. In peer checking, two experienced qualitative researchers continuously evaluated the generated categories and the process of data analysis. Dependability was achieved via the careful documentation of the data analysis and management process, and transferability was achieved by thoroughly describing the study context, including participants’ quotations in the findings, and writing field notes throughout the study. Confirmability was also achieved through triangulation and reflexivity. In triangulation, investigator triangulation was achieved. Multiple researchers independently participated in data analysis and discussed coding decisions to minimize bias and enhance confirmability. Reflective notes were maintained throughout the study to identify and bracket the researchers’ assumptions, experiences, and emotions, helping to minimize potential bias during data analysis and interpretation. The research team included M. Gholami, who had practical experience in a home care center, and nursing faculty members with expertise in medical ethics, home care research, and qualitative methods. All members had extensive experience in nursing ethics, which informed the reflective practice and enhanced the credibility and transparency of the study. An audit trail was maintained throughout the study to confirm transparency and dependability. It included detailed records of data collection, coding, analytic memos, and theoretical sampling decisions. In accordance with the school’s rules, comprehensive reports explaining all research stages were submitted to academic referees every six months, whose feedback was incorporated into subsequent steps. These Persian-language documents provided a clear record of the analytic and methodological decisions made during the research.

### Ethical considerations

The ethical approval for this study was received from the Ethics Committee of Iran University of Medical Sciences, Tehran, Iran (code: IR.IUMS.REC.1401.408). Participants were provided with explanations about the voluntariness of participation and withdrawal and confidentiality of the data. All physical data were stored securely in a locked cabinet accessible only to the study authors. Electronic data were stored on a password-protected computer in encrypted files to ensure confidentiality. Written informed consent was obtained by the researcher (M. Gholami) from all participants.

### Findings

Sixteen HC nurses participated in this study. Most of them were female (*n* = 10) and single (*n* = 12), and had a bachelor’s degree (*n* = 9). The means of their age and work experience were 37.00 ± 5.96 years and 9.43 ± 4.04 years, respectively (Table 1).

**Table 1:** Participants’ characteristics

Participants	Gender	Age (Years)	Marital status	Educational level	Home care experience (Years)
P1*	Female	42	Married	Bachelor’s	16
P2*	Female	49	Married	Bachelor’s	14
P3	Female	34	Single	Bachelor’s	10
P4*	Female	40	Single	Master’s	8
P5	Male	37	Married	Bachelor’s	15
P6*	Male	48	Married	PhD	11
P7	Female	34	Single	Bachelor’s	5
P8	Female	39	Married	Master’s	7
P9*	Female	35	Single	Master’s	8
P10	Male	37	Single	PhD	13
P11*	Female	35	Single	Bachelor’s	9
P12	Female	40	Single	Master’s	12
P13	Male	26	Single	Bachelor’s	2
P14	Male	32	Married	Bachelor’s	6
P15	Female	33	Single	PhD	11
P16	Male	31	Single	Bachelor’s	4

The findings of the study are presented in five main parts, namely main concern, context, strategies, outcomes, and storyline (Table 2).

**Table 2:** The main categories and subcategories of the study

Primary subcategories	Subcategories	Main categories
Lying to the patient to prevent harm; Lying to the patient’s family to promote patient welfare	White-lying	Expedient thinking
Passive/unintentional truth concealing; Active/intentional truth concealing	Truth-concealing	
Making a suggestion and justifying it; Expressing personal experiences; Consulting	Reasoning	Persuading
Trust building; Clarifying	Explaining the advantages of an approach	
Advocating for the patient; Sensitizing; Assertive behavior	Explaining the disadvantages of an approach	
Accepting the risk; Self-justifying to promote patient welfare; Self-justifying to prevent patient harm	Cost-benefit analysis	Self-justifying
Thinking about the outcomes of one’s care practice; Thinking about the outcomes of the patient’s decisions; Thinking about the outcomes of the family’s actions; Analyzing the patient’s living conditions; Thinking about the outcomes of truth-telling	Conditions-consequences analysis	
Reporting the financial exploitation of the patient/family by the home care center; Dissuading the family from requesting unnecessary services; Directly explaining the responsibilities of the colleagues; Informing the patient/family of their ownership of the personally provided equipment	Explicit disclosing	Disclosing
Comparing the effectiveness of medications and equipment; Explaining alternative treatment and care options; Explaining the complications of inappropriate care (Highlighting the necessity of family supervision)	Implicit disclosing	
Facilitating the improvement of the patient’s conditions; The likelihood of legal prosecution; Protecting one’s interests; Colleague-related considerations; Considerations related to the affiliated home care center	The context for expedient thinking	The context for balancing the interests
Endangered health of the patient; Patient’s unrealistic expectations; Patient’s treatment non-adherence; Family’s or physician’s disagreement with the nurse’s care-related suggestions	The context for persuading	
Nurse’s inadequate authority; Patient’s poor prognosis; Coercion	The context for self-justifying	
Limited risk for colleagues; Limited risk for the affiliated home care center; Legal obligations; Hope for receiving divine blessing; The likelihood of harming the patient	The context for disclosing	
Greater precision and caution at work; Improved patient’s and family’s trust in nurses; Greater use of error prevention principles; Sense of worth and satisfaction	Positive outcomes	The outcomes of balancing the interests
Sense of guilt; Anxiety and worry; Sense of inability; Moral distress	Negative outcomes	

### Main concern

Participants’ main concern was fear of harming patients. Patient harm could lead to various consequences for patients, families, and healthcare providers. Therefore, preventing patient harm was the participants’ most important criterion for decision-making.

*The patient had excessive airway secretions and was at high risk of atelectasis and pneumonia due to his immobility and poor clinical condition. However*,* he rejected suctioning and did not allow me to perform the procedure. I felt caught between respecting his autonomy and fulfilling my professional duty to prevent serious harm. I was worried that postponing the intervention could lead to severe complications*,* including infection or respiratory distress. After struggling with this dilemma and considering the potential risks*,* I decided to use a temporary hand restraint to safely perform the suctioning (P.15).*

### Context

Context was one of the most important influential factors on participants’ strategies to manage their main concern. A combination of nurse-related and contextual factors influenced the participants’ strategies to manage their main concern. Nurse-related factors included participants’ personal and professional characteristics as important factors influencing their confrontation with ECs in HC. These characteristics included ethical reasoning orientation, clinical competence, ethical beliefs, professional attitude, interpersonal communication skills, work experience, empathy, compassion, and conscience. Contextual factors encompassed patients’ conditions, families’ socioeconomic status, and inter-professional interactions and relationships (Table 2).

*I was deeply upset when I saw that the patient*,* who could neither speak nor move*,* suffered a burn injury and great pain because my colleague had used the warmer for too long. Reporting the issue could have led to my colleague’s dismissal*,* but I felt torn between loyalty to her and my duty to protect the patient from harm. After reflecting on my responsibility*,* I decided to report the case and reminded myself to always act conscientiously*,* as I would when caring for my own mother (P. 12).*

### Strategies

Participants used four main strategies to manage their main concern. These were expedient thinking, persuading, self-justifying, and disclosing.

### Expedient thinking

Expedient thinking refers to hiding the truth or avoiding honest communication of issues to prevent harm to patients, colleagues, or HC centers. Participants used this strategy when there was a risk of legal prosecution, family members’ negligence toward their patients, and colleagues’ non-professional conduct. This main strategy had two subcategories, namely white-lying and truth-concealing.

#### White-lying

White-lying was an adaptive strategy of participants in their confrontation with conflicting ethical principles, where they had to choose either veracity or patient harm prevention. In this strategy, the non-maleficence principle predominated over the veracity principle. Participants resorted to the white-lying strategy to prevent patient harm when veracity could cause patient harm and when patients’ family members were non-collaborative and negligent towards their patients.

*The results of the complete blood count test showed that the patient’s platelet count was 20,000. However*,* when he asked me about his platelet count after chemotherapy*,* I told him that it had increased to 50*,*000. I felt conflicted because honesty is important to me*,* but I worried that telling him the real number might cause severe distress or anxiety and negatively affect his recovery. I chose to give a more reassuring answer to protect him emotionally*,* although it weighed heavily on my conscience (P. 2).*

#### Truth-concealing

This strategy refers to nurses’ intentional avoidance of providing some information to patients or their families. Our participants used this strategy when they experienced ECs due to the errors of their colleagues or the abusive conduct of the HC center authorities, or when they were worried about legal prosecution or the severe reactions of patients, families, or managers.

*My colleague had injected an analgesic agent for the patient to be able to sleep. The patient had experienced apnea and received mechanical ventilation. Then*,* he needed and underwent a tracheostomy. I felt torn between my ethical responsibility to be honest with the family and my fear that disclosing the event could lead to legal consequences for my colleague and the HC center. The tension between protecting the patient’s right to transparency and safeguarding my colleague’s professional safety placed me in a dilemma. In the end*,* I chose not to inform the family because I was concerned about the potential legal repercussions (P.8).*

### Persuading

This strategy refers to nurses’ attempts to get the agreement of patients, families, and physicians through using their professional knowledge and experience and supporting patients’ rights. Participants used this strategy when they noticed patients’ poor treatment adherence, faced their unrealistic expectations, or had conflicts with families or physicians. This strategy had three subcategories, namely reasoning, explaining the advantages of an approach, and explaining the disadvantages of an approach.

#### Reasoning

Participants attempted to persuade patients, families, or colleagues to accept some specific decisions or actions through explaining their ethical and professional reasons. This strategy was based on interpersonal conversation, discussion, and ethical reasoning. Participants used this strategy particularly when patients, families, or physicians were non-collaborative or there was a risk of patient harm or neglect.

*The physician had prescribed a wet dressing for the patient*,* but based on my professional knowledge and experience*,* I knew it could worsen the wound condition and that a foam dressing was more appropriate. When I explained my reasoning*,* the physician disagreed*,* and the family insisted that the doctor’s order be followed. I felt conflicted between adhering to the physician’s directive*,* respecting the family’s wishes*,* and fulfilling my professional duty to promote the patient’s healing. I explained my reasons for recommending the foam dressing (P.7).*

#### Explaining the advantages of an approach

Another strategy of participants to persuade patients, families, or colleagues was to explain the advantages and positive consequences of a specific decision or approach. They highlighted the practical, mental, or ethical advantages of a specific decision or approach and provided scientific and clinical evidence to reduce existing resistance or disagreement and make better decisions.

*We had a patient with cancer and an extremely low level of albumin*,* yet his family refused to purchase albumin for him. I clearly explained that albumin was essential for reducing edema and improving his clinical condition*,* but they remained unwilling to provide it. This situation placed me in an ethical dilemma: while I felt a responsibility to advocate for the patient’s well-being*,* I also had to respect the family’s decision and their financial limitations. Balancing the patient’s medical needs against the family’s constraints created significant emotional and ethical tension for me (P.4).*

#### Explaining the disadvantages of an approach

In their confrontation with ECs, participants attempted to highlight the potential disadvantages or harms of a decision or action in order to dissuade others. This strategy aimed to prevent harm that could happen due to an inaccurate decision. When a patient’s life was at risk or a medical order or plan was inappropriate, participants attempted to prevent high-risk interventions. They sometimes strongly disagreed with families or HC center authorities or informed patients, families, or physicians of the consequences of a decision or an action in order to protect patients’ rights.

*The patient was in a severe terminal condition with renal failure and a deep coma. His family strongly insisted that we discontinue medical support. Their request placed me in a serious ethical dilemma: while I empathized with their suffering and understood their desire to prevent further hardship for the patient*,* withdrawing life-sustaining treatment is illegal in our country and violates professional regulations. I struggled between respecting the family’s wishes and fulfilling my legal and ethical duty to preserve life. Ultimately*,* I informed them that I could not take responsibility for such an action (P.11).*

### Self-justifying

This strategy refers to nurses’ attempts to justify their unethical practices in order to prevent patient harm, avoid legal prosecution, or be able to perform care measures. Participants used this strategy when they had no other option, had inadequate power to manage the situation, or faced ethical dilemmas. Self-justifying was a cognitive process through which participants attempted to evaluate the situation, weigh the consequences, and use internal justification to persuade themselves that their decisions or actions were the best in that situation. The two subcategories of this category were cost-benefit analysis and conditions-consequences analysis.

#### Cost-benefit analysis

Participants persuaded themselves, based on utilitarianism, deontology, or cost-benefit analysis, that their decisions or actions were beneficial to their patients and had positive consequences despite contradicting some ethical principles. They used this strategy when facing ethical dilemmas or when their patients did not have a good prognosis. This strategy aimed to manage ECs and reduce internal stress.

*The patient was severely agitated*,* and my attempts to calm him verbally were becoming ineffective. I faced a conflict between respecting the patient’s autonomy and ensuring his safety. After careful consideration*,* I decided to use physical restraint to prevent him from removing the catheters and injuring himself (P.9).*

#### Conditions-consequences analysis

In confrontation with ECs, participants attempted to persuade themselves that their decisions were correct through analyzing the objective situation, environmental limitations, and the potential consequences of the decision. The participants described this type of self-justifying as a strategy they used to cope with ECs and to find a rational justification for their decisions in ambiguous or challenging situations. They used this strategy mostly due to families’ coercion and the probability of patient harm. In this strategy, participants analyzed the results of their care measures, consequences of patients’ decisions, results of families’ actions, patients’ living conditions, and consequences of truth-telling.

*The patient’s hemoglobin level had declined to 8. I told them that the patient should be hospitalized due to gastric bleeding; however*,* the patient refused to be transferred. I found myself torn between respecting his autonomy and fulfilling my professional obligation to ensure his safety. Considering the high risk of deterioration and the potential life-threatening consequences of delaying treatment*,* I ultimately decided to transfer him to the hospital despite his disagreement (P.6).*

### Disclosing

This strategy refers to disclosing HC providers’ errors and the financial exploitation of patients and families, as well as helping families make the most appropriate decisions. Participants used this strategy when there was no serious risk to themselves, colleagues, or their affiliated HC center, and when their patients were at great risk for harm. This strategy had two subcategories, namely explicit disclosing and implicit disclosing.

#### Explicit disclosing

In confrontation with ECs, participants directly and explicitly told the truth to patients or their families without any modification, provided that disclosing was not associated with any risk for them, their colleagues, and their affiliated HC center. They performed explicit disclosing based on the veracity, autonomy, and right to know principles. In explicit disclosing, they even disclosed the potential financial exploitation of families by HC centers and dissuaded them from requesting unnecessary services from the centers. Accordingly, they honestly informed patients and families about patients’ conditions and the responsibilities of HC providers. Moreover, they informed patients and families of their ownership of the healthcare equipment that they provided.

*The patient had amyotrophic lateral sclerosis*,* and her condition was progressively worsening. Her family had deliberately withheld information about her diagnosis and prognosis. When she asked me directly to tell her the truth so she could make informed decisions about her life and work*,* I felt caught between respecting the family’s wishes and honouring her right to autonomy and informed decision-making. After struggling with this conflict*,* I chose to be honest with her about her illness and prognosis (P.16).*

#### Implicit disclosing

When the participants found that explicit truth-telling might cause problems for them, their colleagues, or their affiliated HC center, they used indirect and implicit cues and minor information to inform patients and families about some issues. In fact, participants used implicit disclosing, instead of explicit disclosing, to communicate their messages and prevent potential negative consequences (such as distress, isolation, or employment problems). They did this through techniques such as providing comparative information about medication and equipment effectiveness, alternative care and treatment options, treatment-related complications, and the necessity of closer supervision by family members.

*The physician of the HC center had prescribed a BiPAP device*,* while the patient had normal breathing. I informed the patient and their family that this device is usually recommended for those with breathing problems or oxygen desaturation during sleep. I knew that expressing open disagreement with the physician could lead to conflict between me*,* the HC center*,* and the doctor*,* so I chose to indirectly inform them that the device would not be effective for the patient (P.13).*

### Outcomes

The process of the nurses’ confrontation with ECs in HC included experiences that brought about both positive and negative consequences, from the participants’ perspectives. Negative outcomes include guilt, anxiety, worry, moral distress, self-criticism, uncertainty about one’s professional competence, altered mental health, and low care quality. On the other hand, positive outcomes include greater precision and caution at work, improved patients’ and families’ trust in nurses, greater use of error prevention principles, and a sense of worth and satisfaction.

*I knew that the administration of an analgesic by my colleagues had led to drowsiness*,* impaired swallowing*,* and hemodynamic instability. But I didn’t tell the family and the HC center authorities about it. However*,* I still have pangs of conscience due to my silence in that situation (P.10).*

*I explained to the family that weekly laboratory tests were not essential and that the medical order had no scientific justification. Afterward*,* the family decided on monthly laboratory tests*,* and they developed greater trust in me. I felt satisfied with preventing the financial exploitation of the family by the HC center (P.5).*

### Storyline

**Fig. 1:**
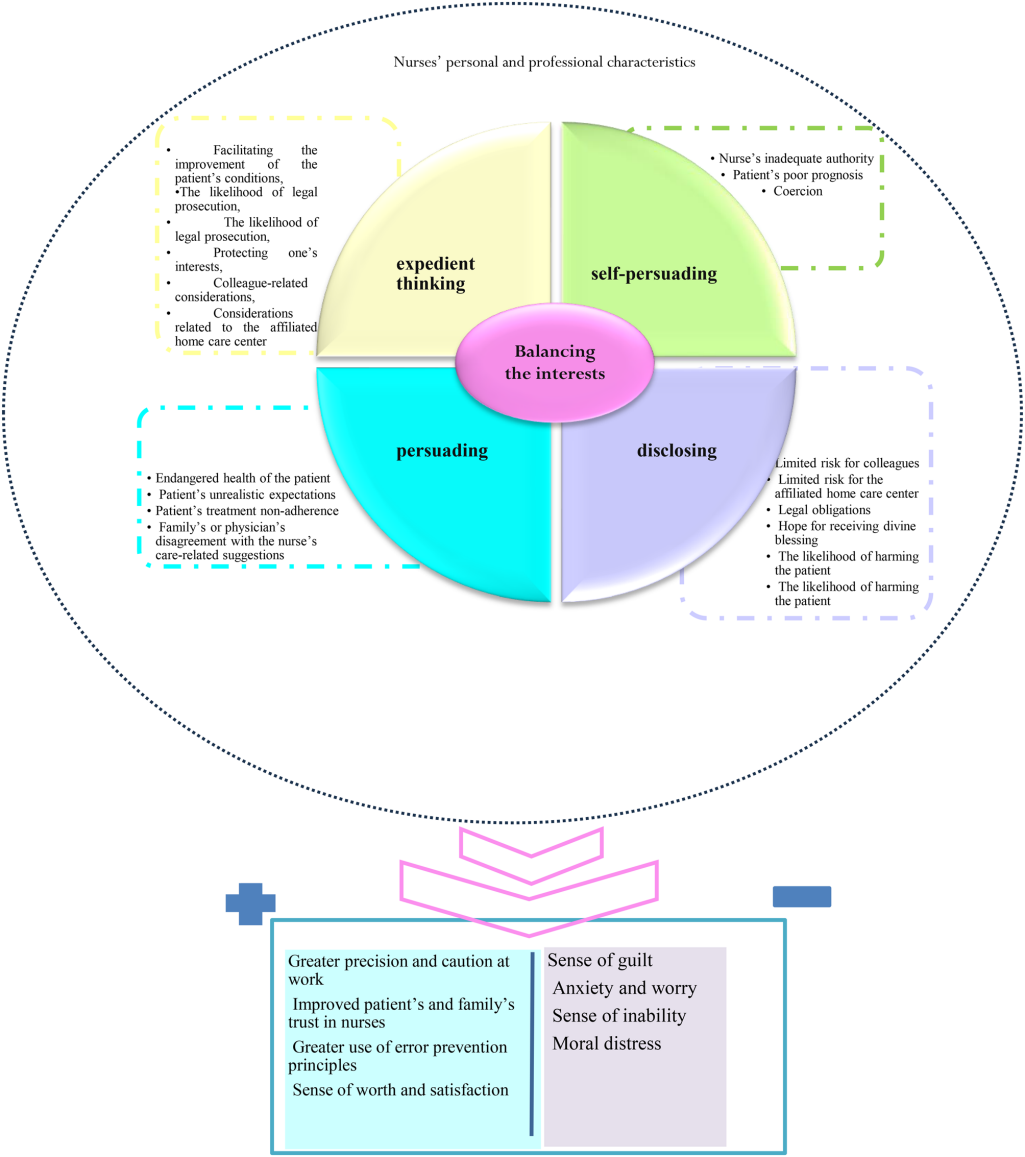
Participants’ process of confronting ethical conflicts in home care

Fear of harming patients was reported by the participants as their main concern when confronting ECs in HC. They reported using strategies such as expedient thinking, persuading, self-justifying, and disclosing in order to manage this concern, balance the interests of themselves, patients, and their families, and prevent legal and professional risks. The participants described attempting to manage ECs in HC with the ultimate goal of preventing harm to patients, families, colleagues, and HC centers. Therefore, the core category of the study is “Balancing interests”. Factors such as nurses’ characteristics, patients’ clinical conditions, families’ socioeconomic status, and interprofessional interactions and relationships shaped how they managed their concerns. From the participants’ perspective, this process sometimes resulted in negative outcomes, such as guilt, anxiety, worry, and moral distress, while also yielding positive outcomes, including greater precision and caution at work, increased patient and family trust in nurses, greater satisfaction, and increased adherence to error prevention strategies. The theory of “Balancing interests”, developed in this study, has a non-linear if-then pattern (Fig. 1).

## Discussion

This study explored the process of nurses’ confrontation with ECs in HC. Findings revealed that the participants use the four strategies of expedient thinking, persuading, self-justifying, and disclosing to balance the interests of all parties involved in the EC situation and prevent harm and risks.

Expedient thinking was one of the strategies used by the participating nurses to manage their ECs in HC. The two subcategories of this strategy were white-lying and truth-concealing. White-lying is a moral decision without any personal motivation, which is used in imbalanced conditions to prevent predictable harm to patients [26]. In such circumstances, it appears that the nurses were more inclined to rely on utilitarianism—prioritizing actions that maximize patient benefit and minimize harm—and virtue ethics, drawing on compassion, benevolence, and practical wisdom. Conversely, deontological principles, which emphasize strict adherence to moral rules and duties, appeared to play a less dominant role in their ethical decision-making when confronted with ECs. A study on pediatric care nurses reported that their use of white-lying depended on the available information, childhood characteristics, family norms, healthcare team competencies, and organizational policies [27]. Another study reported truth-concealing as an ineffective strategy of critical care nurses to reduce their moral distress [28]. Factors such as worry about the legal consequences disclosing errors [29, 30], worry about losing patient trust [29], fear of being accused, fear of managers’ negative reactions, concerns over changes in employment status, and families’ disagreement with truth-telling may cause nurses to resort to truth-concealing, even when truth-disclosing can enhance patient safety [30].

Persuading, through reasoning, explaining the advantages of an approach, and explaining the disadvantages of an approach, was another main strategy of the study participants in their confrontation with ECs in HC. Participants attempted to persuade patients, families, or other HC providers when they faced patients’ treatment non-adherence, patients’ or families’ unrealistic expectations, and families’ or physicians’ disagreement with their care-related recommendations. Persuasion can be a positive intervention when patients or families cannot understand the available risks or when their decisions contradict the long-term goals of care [31]. In agreement with our findings, a study showed that nurses used persuasion to manage ECs when they faced ethical challenges due to patients’ treatment non-adherence [32]. Moral reasoning is a conscious cognitive activity through which an individual weighs an ethical judgment against ethical commitments and principles [33]. HC is associated with specific ethical complexities, limited direct organizational supervision, and great responsibilities for care providers. In HC settings, patients and families expect healthcare providers to respect their treatment- and care-related opinions, desires, and values. Therefore, nurses need advanced communication skills, great ethical awareness, and great sensitivity to patients’ sociocultural context in order to effectively use persuasion, protect patient dignity, and make appropriate care-related decisions [34].

Participants’ other main strategy for managing their fear of harming patients in HC-related ECs was self-justifying, through cost-benefit analysis and conditions-consequences analysis. In agreement with this finding, a study showed that in confrontation with ECs, nurses in Canada attempted to evaluate the consequences of their actions through consulting with their colleagues [16]. Another study in Iran also revealed that nurses analyzed conditions and consequences in ethically difficult conditions [35]. Factors such as patients’ age, comorbid conditions, disease severity, and treatment effectiveness influence healthcare providers’ responses to ethical challenges [36]. Three studies also reported that nurses attempted to balance between the beneficence and the non-maleficence ethical principles while making ethical decisions [17, 32, 37].

Disclosing, both implicit and explicit, was another strategy of participants in their confrontation with ECs in HC. They used this strategy when it was not associated with any negative consequences for themselves, their colleagues, or their affiliated HC centers. Similarly, a study reported disclosing as a main strategy of healthcare providers in their confrontation with ECs regarding information management [36]. Another study revealed that although surgeons honestly explained their errors and unwanted events to their patients, they limited the provided information due to their legal or professional concerns [38]. Disclosing is far beyond communicating errors and is considered a multidimensional, ethical, and well-organized strategy for managing ECs, respecting patient rights, and creating a trusted care setting [29]. Appropriate timing and adequate clarity in disclosing win patient and family trust, reduce concealing-related conflicts [39], and indicate nurses’ commitment to provide comprehensive care and protect patients’ human dignity [29]. A preventive and prospective approach to disclosing improves care quality and builds trust in care-related relationships [39]. Moreover, a supportive organizational climate and a facilitating culture increase the probability of disclosing unethical behaviors, while a fearful non-confident climate increases the probability of ethical silence and truth-concealing [40].

### Strengths and limitations

This study had several strengths. The grounded theory design helped provide a comprehensive and contextualized understanding of nurses’ confrontation with ECs in HC. The researcher’s reflexivity and continuous supervision by an expert team further supported the trustworthiness of the analysis. Moreover, the developed theory offers valuable insights that can inform ethical nursing practice and policy in home care settings. Some limitations must be taken into account when interpreting the study findings. First, the data were collected solely from HC nurses, and the findings may not reflect the experiences and perspectives of patients, families, or healthcare managers. Second, the high sensitivity of the EC subject matter might have prevented some participants from sharing all aspects of their EC-related experiences. We attempted to mitigate this limitation using various interview questions. Third, this qualitative study was conducted in the sociocultural context of Iran, and the transferability of its findings to other settings should be performed cautiously.

## Conclusion

This study concludes that the process of nurses’ confrontation with ECs in HC is the process of “Balancing interests” using the four main strategies of expedient thinking, persuading, self-justifying, and disclosing. This process starts with the main concern of fear of harming patients and aims to balance between the interests of all parties involved in the EC situation.

This study offers a comprehensive understanding of how homecare nurses encounter ECs in their practice. The findings provide valuable insights aimed at enhancing ethical decision-making, improving patient safety, and guiding the development of training programs and policies that support nurses in homecare settings. By emphasizing the challenges faced by nurses and the strategies they employ, this research contributes to the advancement of clinical practice and nursing education on a global scale.

### Recommendations for future research and education

Based on the findings of this study, further research is recommended to explore the experiences of other stakeholders involved in HC, such as patients, family caregivers, and physicians, to gain a more comprehensive understanding of ECs in this context. Future studies could also investigate the effectiveness of interventions designed to enhance nurses’ ethical sensitivity, moral reasoning, and coping strategies when facing ethical challenges in home settings. From an educational viewpoint, the development of continuing education programs and ethics training modules tailored to home care practice is essential. Integrating ethical reflection sessions and interdisciplinary workshops into nursing education could help prepare nurses to address ECs more confidently and competently in HC settings.

### Implications for practice

Nursing managers and policymakers can utilize the findings of this study to recognize ethical conflicts in home care better, understand how nurses address them, and develop effective strategies to promote ethical care delivery and foster supportive work environments. Implementing such strategies may help reduce nurses’ moral and mental workload, enhance the quality of nursing care, and ultimately improve patient outcomes.

## Supplementary Information

Below is the link to the electronic supplementary material.

Supplementary Material 1

## Data Availability

If requested, the authors who correspond with us will gladly share the datasets that were utilized and analyzed in the course of this research study.

## Abbreviations

ECs: Ethical conflicts
HC: Home care
