# Supplementary material for: The process of nurses’ confrontation with ethical conflicts in home care: a grounded theory study
Source: BMC Nurs. 2025 Dec 22;24:1483. doi: 10.1186/s12912-025-04195-2 (PMC12720444; doi:10.1186/s12912-025-04195-2)
Supplement: Supplementary file 1 — Supplementary Material 1 [file 12912_2025_4195_MOESM1_ESM.docx]

**Interview Guide – Supplementary File**

Title: Interview Guide Used in the Study “The Process of Nurses’ Confrontation with Ethical Conflicts in Home Care: A Grounded Theory Study”

**Introduction for Participants:**

Greetings, and thank you for your time.

I would like to conduct an interview with you as part of my thesis research. The purpose of this study is to explain the process of nurses’ confrontation with ethical conflicts in home care.

The questions in this interview are related to your experiences with ethical conflicts during home care. Your responses will be used only for research purposes.

Your name and personal information will remain completely confidential, and no identifying details will appear in the thesis or any related publication.

Please note that your participation is voluntary, and you may withdraw from the interview at any time without any consequences.

**Main Interview Questions**

1. Please tell me a little about your work experience in home care.
2. What types of patients do you usually care for?
3. What do you typically do during a working day when caring for a patient at home?
4. Please describe your experiences of facing situations where you had to choose between different ethical principles.
5. What issues have you observed in the home environment of patients that lead to ethical conflicts?
6. How do you react when you see colleagues providing care that seems unethical?
7. How did you feel in those conflict situations?
8. What did you do when you were uncertain about the ethical correctness of a care-related action or behavior?
9. What consequences have these situations had for you (professionally, psychologically, morally, or in your job)?
10. What strategies or actions have you used to manage or resolve these ethical problems?
11. What factors (personal, organizational, family, cultural) influence your decisions in such situations?
12. What was the outcome of your actions (for you, the patient, the family, and the home care organization)?
13. Is there any other issue related to ethical conflicts in home care that I have not asked about and that you think should be discussed?

**Probing Questions**

Could you please explain more about that?

What do you mean exactly?

Could you give a specific example based on your experience?

**Closing Remark**

Thank you very much for sharing your valuable experiences with me
